# 

**Published:** 1899-07-29

**Authors:** 


					s\y THE HOSPITAL. " The standard of highest purity."? Lancet. July 29th, 1899.
CADBURY'S r? U fc* OADBURY'S
COCOA m> JKH> COCOA
ABSOLUTELY PURE. ^ NO DRUGS OR ALKALIES USED.
\X\
A
9i
^Glasco? Western Int^rmahy'
No. 67f Vol. XXVI. Saturday,
July 29th, 1899.
iriciTksspjTji a
rSubsoription Terms,"]
boo p. 292. J
PRICE TWOPENCE.

				

